# Pathological frataxin deficiency in mice causes tissue-specific alterations in iron homeostasis

**DOI:** 10.1016/j.isci.2025.114625

**Published:** 2026-01-05

**Authors:** Maria Pazos-Gil, Marta Medina-Carbonero, Arabela Sanz-Alcázar, Marta Portillo-Carrasquer, Luiza Oliveira-Jorge, Gonzalo Hernández, Mayka Sánchez, Fabien Delaspre, Elisa Cabiscol, Joaquim Ros, Jordi Tamarit

**Affiliations:** 1 Departament Ciències Mèdiques Bàsiques, Facultat Medicina, IRBLleida, Universitat de Lleida, Lleida, Spain; 2 Iron Metabolism: Regulation and Diseases Group, Department of Biomedical Sciences, Universitat Internacional de Catalunya (UIC), Sant Cugat del Vallès, Spain

**Keywords:** health sciences

## Abstract

Friedreich ataxia is caused by partial frataxin deficiency due to genetic mutations. It is well established that frataxin knockout affects iron homeostasis, but the alterations caused by pathological (partial) frataxin deficiency are poorly understood. In this study, we have analyzed iron homeostasis in a mouse model presenting pathological frataxin deficiency (FXNI151F). Our results reveal tissue-specific alterations of iron regulatory proteins (IRPs). In the heart, IRP2 accumulation is observed, likely triggered by iron-sulfur deficiency, while IRP1 is decreased in the cerebellum and liver. We also found elevated iron levels in mutant mice. Accumulation was particularly pronounced in the cerebellum, where increases were already evident at 10 weeks. Hepatic accumulation was not manifested until 21 weeks and was more pronounced in females. Overall, these findings indicate that frataxin deficiency disrupts iron homeostasis in a tissue-, age-, and sex-dependent manner, and provide novel insights into the mechanisms causing these perturbations.

## Highlights

• FXNI151F mice present partial frataxin deficiency, as Friedreich ataxia patients
• FXNI151F mice show tissue and sex-specific alterations in iron homeostasis
• Heart presents IRP2 accumulation, Tfrc induction, and ferritin decrease
• Cerebellum and liver present iron accumulation and IRP1 content decrease

## Graphical abstract

**Figure:**
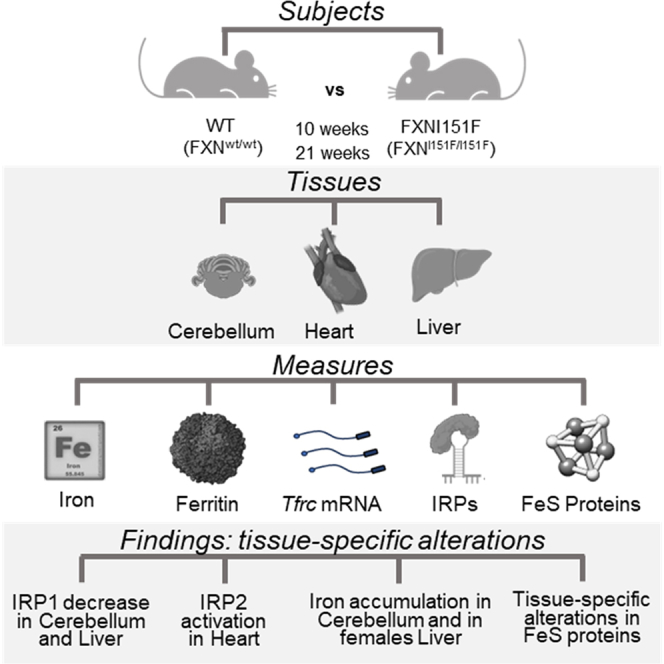

## Introduction

Friedreich ataxia (FA) is an inherited recessive disease caused by mutations in the frataxin gene (*FXN*). The most common mutation is a GAA triplet expansion in the first intron of *FXN*, causing a systemic partial deficiency in frataxin protein expression.^1^ Around 4% of patients are compound heterozygous for a GAA expansion and a *FXN* point mutation or deletion.^2^ Clinical manifestations are mostly observed in the nervous system and the heart.^3^ Frataxin is predominantly localized in the mitochondria, where it can function as a kinetic accelerator of persulfide transfer between cysteine desulfurase (NFS1) and iron-sulfur cluster assembly enzyme (ISCU), a critical step in the synthesis of iron-sulfur clusters.^4^^,^^5^^,^^6^ Its deficiency causes dysregulation of iron homeostasis, with iron overload first described in frataxin-deficient budding yeast.^7^ Subsequent studies with this organism indicated that frataxin deficiency caused an anomalous activation of the iron regulon.^8^^,^^9^ Alterations in iron homeostasis have also been observed in patients and mammalian models of FA.^10^^,^^11^^,^^12^ Regarding mice, iron aggregates were described in heart mitochondria from conditional cardiac/skeletal muscle frataxin knockout (KO) mice (MCK mice).^13^^,^^14^ These mice also presented changes in the expression of proteins involved in cellular iron homeostasis, such as transferrin receptor 1 (TFR1; gene *Tfrc*) or ferritins, as well as activation of iron regulatory protein 2 (IRP2). Elevated IRP2 activity was also observed in cultured fibroblasts derived from FA patients.^15^ Frataxin deficiency can also affect iron regulatory protein 1 (IRP1, also termed ACO1), a protein that can be found in holo (with iron-sulfur cluster and aconitase activity) and apo (mRNA-binding) forms.^16^ A marked deficiency in ACO1/IRP1 content was reported in liver-specific FXN KO mice^17^ and in the MCK mouse.^18^ While iron-mediated regulation of IRP activity is well characterized (reviewed in Galy et al.^16^), the mechanisms causing IRPs alterations in frataxin-deficient mammals are not completely understood. It is presumed that these alterations are a consequence of deficient mitochondrial iron-sulfur biogenesis. Thus, prolonged loss of iron-sulfur clusters would result in decreased stability of ACO1/IRP1,^18^ while IRP2 content would increase, since its degradation is mediated by the ubiquitin ligase FBXL5, which also contains an iron-sulfur cluster.^19^ Nevertheless, as iron-sulfur deficiency is not always observed in frataxin-deficient models,^20^^,^^21^ the involvement of other pathways in IRPs activation or destabilization should not be excluded.

As indicated above, alterations in iron homeostasis in mice have been mostly explored in conditional mouse models presenting null frataxin levels in the targeted tissues and normal expression in all the others. Although the results obtained in these models have contributed to understanding the consequences of frataxin deficiency on iron metabolism, these conditional models do not fully replicate FA, since patients exhibit low frataxin levels across all tissues. We recently generated a mouse model based on the human pathological point mutation I154F, which affects frataxin stability and has diminished capacity to stimulate the cysteine desulfurase reaction *in vitro.*^22^ Mice homozygous for this mutation (FXNI151F) present low frataxin levels in all tissues and display weight loss, neurological deficits, and biochemical alterations.^23^^,^^24^ This model does not carry the GAA expansions found in most patients, but it exhibits low frataxin levels and alterations that closely resemble those observed in patients. Consequently, it represents an excellent model for investigating the tissue-specific consequences of pathological frataxin deficiency (defined as a systemic partial deficiency, as found in FA patients). In this regard, we recently analyzed iron homeostasis parameters and ferroptosis markers in dorsal root ganglia isolated from this model, finding increased TFR1 expression, decreased ferritin heavy chain (H-FT) content and increased mitochondrial iron.^25^ In the present work, we have explored the alterations in iron homeostasis in the cerebellum, heart, and liver, concluding that iron homeostasis alterations in FXNI151F mice are tissue-, age-, and sex-specific. These findings are crucial for understanding the consequences of pathological frataxin deficiency on the tissues affected by this disease.

## Results

The main objective of this work was to analyze the alterations in iron homeostasis in the cerebellum, heart, and liver caused by pathological frataxin deficiency. The first two organs were chosen because they are notably affected in FA patients. The liver was selected because it plays a key role in systemic iron metabolism. To gain a clearer view of the evolution of the analyzed parameters, we conducted the study at 10 and 21 weeks of age. It should be noted that FXNI151F mice begin to exhibit neurological alterations around 20 weeks, while loss of weight gain is already evident from 15 weeks onwards.^23^ The analyzed parameters were frataxin levels, iron content, IRPs 1 and 2, the protein content of ferritin light (FT-L) and heavy (FT-H) chains, and *Tfrc* mRNA levels. It is worth reminding that *Tfrc* and ferritins are post-transcriptionally regulated by IRPs through binding to iron regulatory elements (IREs) present in their mRNAs. *Tfrc* mRNA presents IREs at 3′ from the coding region and IRP binding protects it from degradation. In ferritins, IREs are at 5′ from the coding region and IRP binding inhibits their translation. Therefore, IRP activation increases *Tfrc* mRNA levels and decreases ferritins protein content.^26^ We also analyzed aconitase 1 and 2, both content and activity, as an indicator of iron-sulfur proteins status.

### The cerebellum of FXN151F mice exhibits iron accumulation and decreased IRP1 content

**Figure 1:**
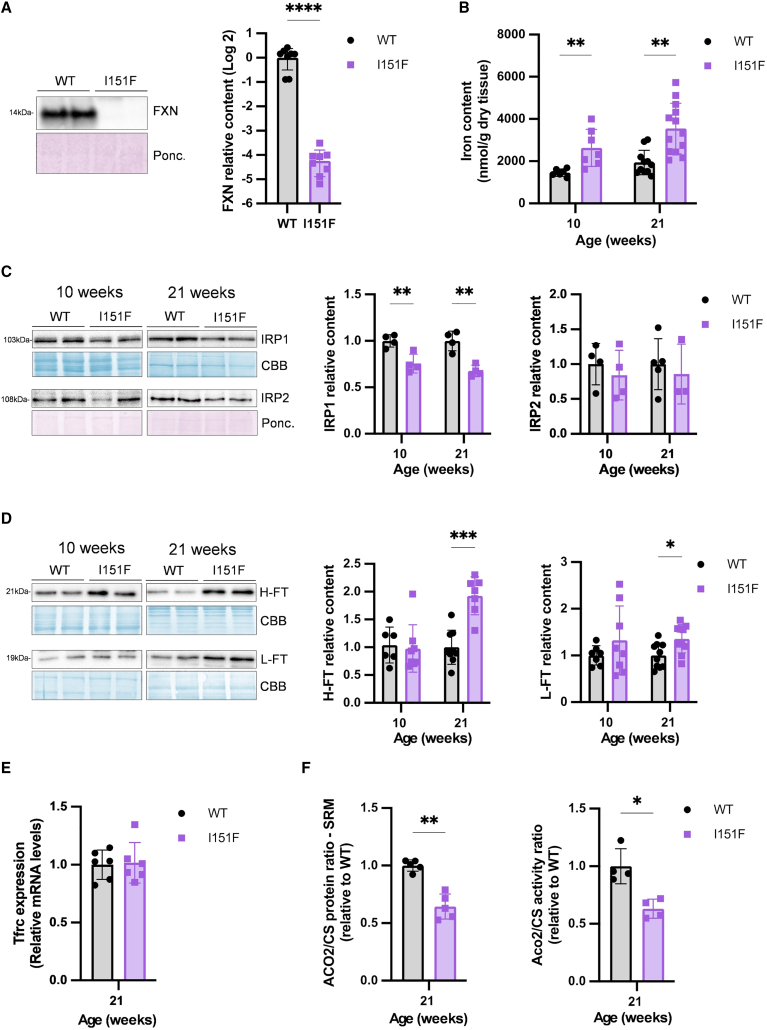
Consequences of frataxin deficiency in the cerebellum For proteins assessed by western blot, representative images are shown, while histograms represent the relative protein content calculated from the western blot signal normalized to the CBB or Ponceau stain. Data are presented as the mean ± SD, symbols correspond to biological replicates. Significant *p* values are indicated as <0.05(∗), <0.01(∗∗), <0.001(∗∗∗), <0.0001(∗∗∗∗). Differences between groups in iron content were assessed by two-way ANOVA with Tukey’s multiple comparison test. Two-tailed Student’s *t* test was used to assess the significance between WT and I151F mice in all other data presented. (A) Frataxin levels in 10-week-old mice. (B) Non-heme iron levels from WT and FXNI151F mice at indicated ages. (C) ACO1/IRP1 and IRP2 protein content measured by western blot. (D) H-FT and L-FT ferritins measured by western blot. (E) *Tfrc* mRNA content measured by quantitative reverse-transcription PCR (RT-qPCR). (F) Aconitase 2 to citrate synthase ratios (ACO2/CS). Relative protein ratio was calculated by SRM-proteomics, while activity ratio was calculated spectrophotometrically.

We confirmed that the cerebellum from 10-week-old FXNI151F mice presented low frataxin levels by western blot (approximately 4% of wild-type [WT] levels) (Figure 1A). Similar frataxin values had previously been described in these mice at 21-weeks of age.^23^ To explore the consequences of frataxin deficiency on cerebellum iron homeostasis, we first analyzed iron levels. As shown in Figure 1B, increased iron content was observed in FXNI151F cerebellum at 10- and 21-weeks of age. We next analyzed IRP1 and IRP2 content by western blot. We observed decreased content of IRP1, while no differences were detected in IRP2 content (Figure 1C). We also measured the *Tfrc* expression by qPCR, and ferritins content by western blot. We observed increased FT-H and FT-L content in 21-week-old FXNI151F mice, while no changes were observed in *Tfrc* mRNA levels (Figures 1D and 1E). Aconitase activities and content were also measured. This required setting up the assays, as mammalian cells contain two aconitase isoenzymes: ACO1, which is the iron-sulfur containing proteoform of IRP1 and is located in the cytosol, and aconitase 2 (ACO2), which is mitochondrial. According to data from the PaxDb database,^27^ ACO2 accounts for nearly 90% of total aconitase content in the brain and heart, while both isoenzymes are similarly expressed in the liver. To confirm these estimations, we first measured aconitase activity in the cerebellum, heart, and liver from 21-week-old WT mice by an in-gel activity assay, in which the two isoenzymes present different migration (Figure S1A). By immunoblot detection, we identified the lower bands as ACO1 and the upper band as ACO2. We also observed that ACO1 activity was barely detected in the cerebellum and heart, which is consistent with the reported abundance data of these proteins in the PaxDb database. We concluded that ACO1 activity could not be reliably detected in the cerebellum nor in the heart and that its contribution to total cellular aconitase activity in these organs was minimal. Therefore, for measuring ACO2 activity in cerebellum and heart, we used tissue homogenates and a spectrophotometric assay, which allows the normalization of aconitase activity to citrate synthase (CS) activity (used as an indicator of total mitochondrial activity). Using this assay, we observed a 37% decrease in ACO/CS activity in 21-week-old FXNI151F mice (Figure 1F). To determine if this decrease was caused by lower ACO2 protein content, or by decreased enzyme-specific activity, we measured the relative ACO2/CS protein content ratio by selected reaction monitoring (SRM)-mass spectrometry, which provides accurate quantitation and allows the comparison of ratios between different proteins across multiple samples (representative SRM traces are shown in Figures S1B and S1C). Using this approach, we observed that the ACO2/CS protein content ratio was 36% decreased (Figure 1F). Therefore, we concluded that specific ACO2 activity (estimated by dividing activity by protein ratios) was not altered in FXNI151F cerebellum.

### IRP2 is activated in the heart of the FXN151F model

**Figure 2:**
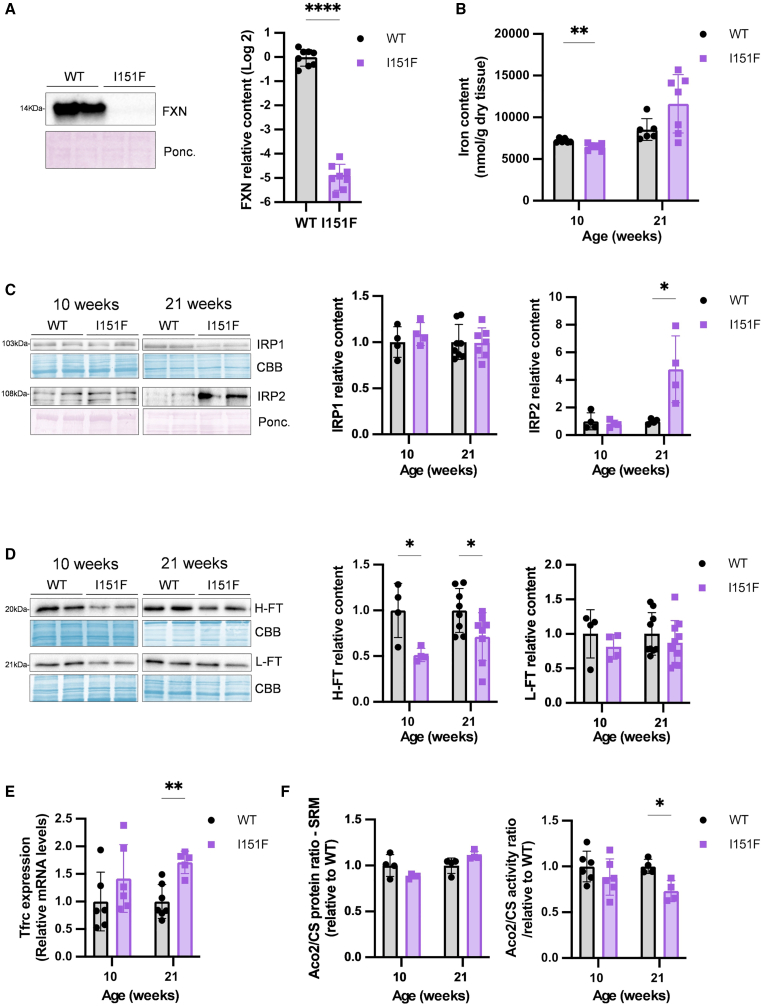
Consequences of frataxin deficiency in the heart Data are presented as the mean ± SD, symbols correspond to biological replicates. Significant *p* values are indicated as <0.05(∗), <0.01(∗∗), <0.0001(∗∗∗∗). Differences between groups in iron content were assessed by two-way ANOVA with the Tukey’s multiple comparison test. Two-tailed Student’s *t* test was used to assess the significance between WT and I151F mice in all other data presented. (A) Frataxin levels measured by western blot in 10-week-old mice. (B) Non-heme iron levels from WT and FXNI151F mice at indicated ages. (C) ACO1/IRP1 and IRP2 protein content measured by western blot. (D) FT-H and FT-L ferritins measured by western blot. (E) *Tfrc* mRNA content measured by RT-qPCR. (F) Aconitase 2 to citrate synthase ratios (ACO2/CS). The relative protein ratio was calculated by SRM-proteomics, while the activity ratio was calculated spectrophotometrically.

We performed a similar analysis in heart. Frataxin levels in 10-week-old FXNI151F mice were ≈4% of those observed in WT animals (Figure 2A). A slight increase in iron content was observed in 21-week-old FXNI151F mice, without reaching statistical significance (Figure 2B). Regarding IRP levels, we observed increased content in IRP2 in 21-week-old FXNI151F mice, but not in 10-week-old animals. No changes were observed in IRP1 content (Figure 2C). FT-H levels decreased, both at 10 and 21 weeks (Figure 2D), while *Tfrc* mRNA levels increased, particularly at 21 weeks (Figure 2E). These results indicate that IRP2 is abnormally activated in the heart of the FXN151F model. IRP2 accumulation has been detected in other FA models, and it has been suggested that this could be due to a depletion of cytosolic iron caused by the accumulation of this metal in the mitochondria.^28^ Therefore, we obtained cytosolic and mitochondrial fractions from heart tissue (10- and 21-week-old animals) and measured their iron content using inductively coupled plasma mass spectrometry (ICPMS). Nevertheless, we did not find any significant difference in mitochondrial nor cytosolic iron between WT and mutant animals (Figure S2). These results are in line with previous studies that did not report reduced cytosolic iron content in FA models.^11^^,^^15^^,^^29^ Therefore, in our model, IRP2 accumulation in the heart cannot be attributed to a decrease in cytosolic iron. Another possibility that has been suggested is that IRP2 accumulation may be caused by a deficiency in iron-sulfur clusters, which are involved in the regulation of the iron deficiency response. Therefore, we measured aconitase activity and content (using the same approach described for cerebellum) and we found that aconitase activity was 27% decreased in 21-week-old FXNI151F animals (Figure 2F). No changes were observed in ACO2/CS protein ratio, indicating that loss of aconitase activity was caused by decreased ACO2 specific activity. This point to a deficit in the biogenesis of iron-sulfur clusters or to a lack of iron bioavailability as possible causes of decreased activity of this enzyme, and the consequent activation of IRP2.

### Sex-specific iron homeostasis alterations are found in the liver

**Figure 3:**
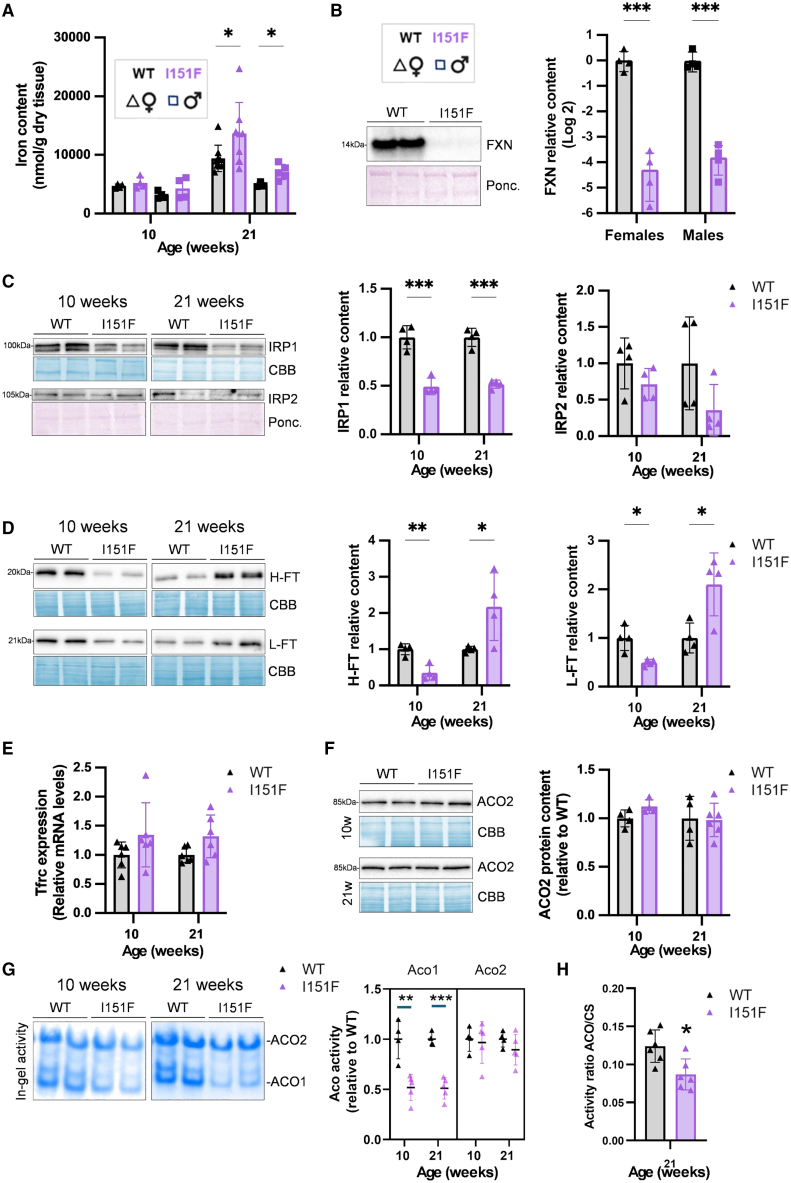
Consequences of frataxin deficiency in liver Data are presented as the mean ± SD, symbols correspond to biological replicates (squares to males, triangles to females). Significant *p* values are indicated as <0.05(∗), <0.01(∗∗), <0.001(∗∗∗). Differences between groups in iron content were assessed by two-way ANOVA with the Tukey’s multiple comparison test. Two-tailed Student’s *t* test was used to assess the significance between WT and I151F mice in all other data presented. (A) Non-heme iron levels from WT and FXNI151F mice, females, and males, at indicated ages. (B) Frataxin levels measured by western blot in 10-week-old mice, in both females and males. (C) ACO1/IRP1 and IRP2 protein content measured by western blot in females. (D) FT-H and FT-L ferritins measured by western blot in females. (E) *Tfrc* mRNA content measured by qPCR in females. (F) ACO2 content measured by western blot in females. (G) Aconitase 1 and 2 activities measured by in-gel activity assay in females. (H) Aconitase to citrate synthase ratio calculated spectrophotometrically in livers from 21-week-old females.

When we analyzed the iron content in the liver, we noticed that at 21 weeks of age, it was higher in females than in males. This difference was more pronounced in the mutants, which showed higher iron levels than the WT (Figure 3A). In the cerebellum and heart, these differences between sexes were not observed (Figure S3). Increased liver iron content in females had previously been described in WT mice^30^ and in murine models of hemochromatosis,^31^ indicating that female mice are more prone to accumulate iron than males. Frataxin content in livers from I151F animals was ≈4% compared to WT animals, in both females and males (Figure 3B). Due to the differences in iron accumulation between males and females, we decided to analyze livers from both sexes separately. Results from females are shown in Figure 3, and those from males in Figure S4. In females, IRP1 content was decreased, while no significant differences were found in IRP2 content, indicating absence of activation of this protein (Figure 3C). Both ferritins presented age-dependent alterations, a decrease in content in FXNI151F mice at 10 weeks followed by an increase at 21-weeks (Figure 3D). *Tfrc* expression showed a slight increase, which was only significant in 10-week-old males (Figures 3E and S4). Regarding aconitases, we used western blot for assessing ACO2 content (Figure 3F) and the in-gel activity assay for assessing ACO1 and ACO2 activities (Figure 3G). We observed decreased ACO1 activity (≈50%) in FXNI151F mice, while no significant differences were observed in ACO2 activity nor content. The decrease in ACO1 activity (Figure 3G) was similar to the decrease in ACO1/IRP1 quantity detected through western blot (Figure 3C). This suggests that the specific activity of this enzyme was not markedly altered. To further validate these activity analyses, we measured total aconitase activity spectrophotometrically in 21-week-old mice. By this assay, we observed a 30% decrease in the mutants (Figure 3H), a value consistent with the results obtained with the in-gel assay as total aconitase activity in liver is similarly contributed by both isoenzymes. We next analyzed these parameters in males (Figure S4). We observed a similar behavior, but the differences between WT and mutants were less pronounced than those found in females, especially in ACO1 activity.

### Investigating the causes of ACO1/IRP1 deficiency in liver: Iron-sulfur protein status

**Figure 4:**
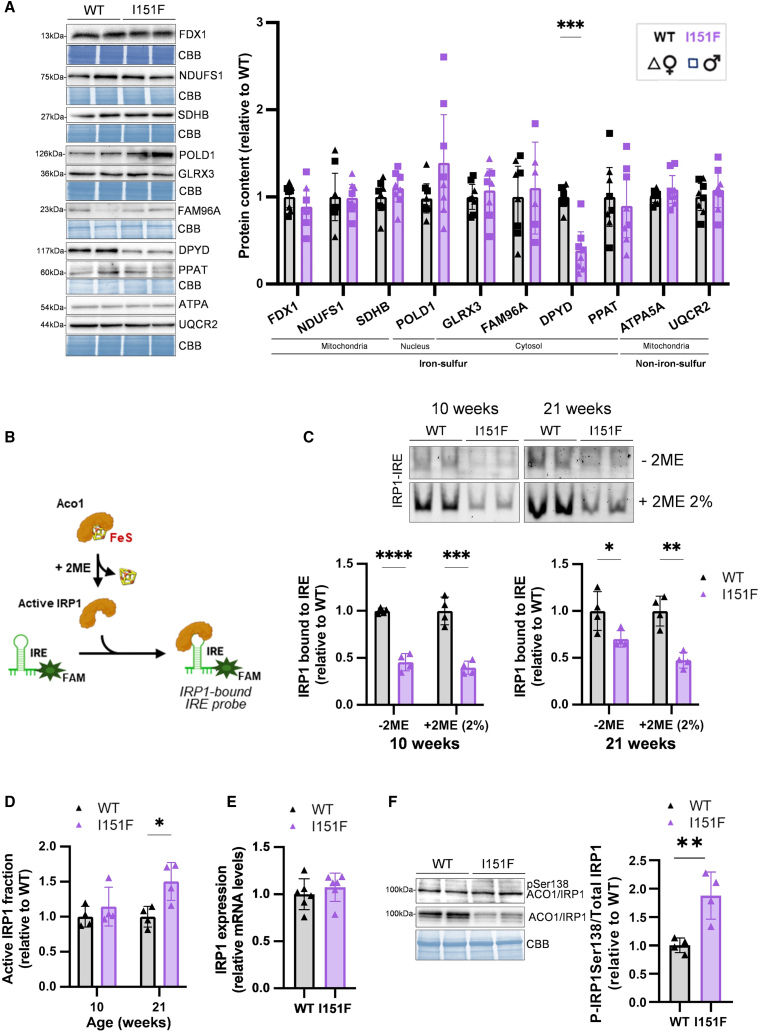
Investigating the causes of ACO1/IRP1 deficiency in livers from 10-week-old mice Data are presented as the mean ± SD, symbols correspond to biological replicates (squares to males, triangles to females). Significant *p* values are indicated as <0.05(∗), <0.01(∗∗), <0.01(∗∗∗), <0.0001(∗∗∗∗). Two-tailed Student’s *t* test was used to assess the significance between WT and I151F mice. (A) Iron-sulfur protein status: representative western blot images and quantitative data from the indicated proteins. (B) Diagram indicating the principle of the EMSA assay used to analyze the activation level of IRP1. Iron-sulfur cluster loss or 2 ME treatment activates IRP1, which can bind to a fluorescent IRE-containing mRNA probe and change its electrophoretic mobility. (C) EMSA analysis from female liver samples. Representative assays showing the band corresponding to the IRP1-bound IRE mRNA probe (IRP1-IRE), in the presence and absence of 2 ME. (D) Quantification of the IRP1-IRE band and estimation of the active IRP1 fraction (relative to WT) found in livers of 10- and 21-week-old females. (E) ACO1/IRP1 mRNA expression measured by qPCR in livers from 10-week-old females. (F) Detection of total ACO1/IRP1 and a phosphorylated proteoform (pS138IRP1) by specific antibodies. The histogram represents the relative quantification of the pIRP1/total IRP1 ratio.

ACO1/IRP1 deficiency has previously been described in several FA models^17^^,^^18^ and attributed to decreased iron-sulfur cluster biogenesis. However, the lack of inactivation of liver mitochondrial ACO2 suggests that mitochondrial iron-sulfur cluster biogenesis is not strongly affected. To obtain a more comprehensive picture of iron-sulfur cluster status in the liver, we evaluated the content of three additional mitochondrial proteins that contain iron-sulfur centers: NDUFS1 (complex I subunit), SDHB (complex II subunit), and ferredoxin 1 (FDX1). We also measured two mitochondrial non-iron-sulfur proteins as controls, UQCRC2 subunit from complex III and the ATPA subunit from complex V. None of these mitochondrial proteins were altered in FXNI151F mice (Figure 4A). We next focused on the cytosolic iron-sulfur assembly pathway (CIA), as ACO1/IRP1 deficiency could also be caused by impairment in this pathway. With this purpose, we evaluated the content of glutaredoxin-3 (GLRX3), which plays a general role in intracellular iron supply^32^ and whose depletion decreases the content and/or activity of several extramitochondrial iron-sulfur proteins, including IRP1.^33^ We also evaluated amidophosphoribosyltransferase (PPAT), dihydropyrimidine dehydrogenase (DPYD) and the catalytic subunit from DNA polymerase delta 1 (POLD1), each of them requiring a different CIA subcomplex for its maturation. FAM96A (CIA2A), a member of the CIA machinery involved in IRP1 maturation, was also analyzed. We observed decreased content of DPYD in FXNI151F mice, more marked in females (Figure 4A). However, none of the other proteins analyzed were decreased, confirming that partial frataxin deficiency does not cause a general loss in iron-sulfur proteins. Overall, we can conclude that ACO1/IRP1 decrease in 10-week-old liver is not caused by impairment of the mitochondrial or cytosolic iron-sulfur maturation pathways.

### Partial iron-sulfur cluster loss may contribute to ACO1/IRP1 deficiency in the cerebellum and in 21-week-old liver

To further analyze if ACO1 was losing its iron-sulfur cluster in FXNI151F mice, we conducted an electrophoretic mobility shift assay (EMSA). This assay detects the presence of active IRP1 by analyzing its binding to an IRE-containing mRNA probe. The EMSA assay was first performed using liver homogenates from females, as expression of ACO1/IRP1 is higher in the liver than in the cerebellum, and because females showed a more pronounced decrease in both ACO1/IRP1 content and ACO1 activity compared to males. As a positive control, cellular extracts were treated with 2-mercaptoethanol (2-ME) to fully activate IRP1 (Figure 4B). We observed that the amount of active IRP1 (IRP1-IRE band) was decreased in FXNI151F mice, both in the presence or in the absence of 2-ME, at 10 and 21 weeks of age (Figure 4C). The lower amount of IRP1-IRE band observed in mutant mice in the positive control (+2-ME) was consistent with the lower ACO1/IRP1 content detected by western blot in these animals (Figure 3C). Moreover, to estimate the proportion of ACO1/IRP1 in its active IRP1 form (bound to IRE), we compared the IRE-bound IRP1 values obtained in the absence of 2-ME with those obtained in the presence of this compound. This calculation indicated that the proportion of protein in active IRP1 form was not altered in 10-week-old animals, but it was slightly increased in 21-week-old mice (Figure 4D). This result indicates that ACO1/IRP1 decrease at 10 weeks is not caused by a loss of its iron-sulfur center. Nevertheless, this phenomenon could occur at later ages, as suggested by the increased proportion of active IRP1 in 21-week-old mice. We next performed the same analysis in 10-week-old cerebellum, which is the other tissue where ACO1/IRP1 content was decreased. In this case, we observed results similar to those obtained in 21-week-old liver (Figure S5). Therefore, ACO1 could be experiencing a partial loss of its iron-sulfur cluster in cerebellum, and its deficiency triggered by this loss. However, in none of the conditions analyzed, did we observe an increase in the active form of IRP1, indicating that in no case was the partial activation of this protein sufficient to compensate for the decrease in its content.

### ACO1/IRP1 is phosphorylated in liver of 10-week-old FXNI151F mice

Our results suggest that partial iron-sulfur cluster loss could be contributing to ACO1/IRP1 protein deficiency in the cerebellum and in 21-week-old liver. Nevertheless, other mechanisms may also be involved in this deficiency, since in 10-week-old livers, no activation of IRP1 was observed, indicating that this protein had not lost the iron-sulfur center. Moreover, as we have shown above, at this age we did not observe a general decrease in proteins containing this cofactor. To further explore potential mechanisms contributing to ACO1/IRP1 loss in 10-week-old liver, we measured its mRNA expression by qPCR. We did not observe any decline in mRNA expression, indicating that protein loss is caused by post-transcriptional events (Figure 4E). Since phosphorylation of this protein at Ser-138, has been linked to increased sensitivity to degradation,^34^ we decided to explore the ACO1/IRP1 phosphorylation status using phospho-Ser138 antibodies. As shown in Figure 4F, the intensity of the phospho-Ser138 band was similar between WT and FXNI151F mice. As total ACO1/IRP1 is decreased, we can conclude that increased phosphorylation of this residue is occurring in the livers of FXNI151F mice. Therefore, our results suggest that ACO1/IRP1 phosphorylation may be contributing to its degradation in frataxin-deficient liver.

## Discussion

The consequences of frataxin deficiency on iron metabolism had previously been analyzed in heart or liver-specific KO frataxin mice.^13^^,^^17^ However, these conditional models did not accurately replicate the human pathological condition, as patients present partial frataxin deficiency in all the tissues. By contrast, most murine models based on GAA expansions fail to reach sufficiently severe frataxin deficiencies to elicit pronounced functional and biochemical alterations, thereby limiting their utility for studies of this nature (reviewed in Mosbach and Puccio^35^). This phenomenon likely reflects the fact that a more profound frataxin deficiency is required in mice than in humans to produce comparable effects. In the present work, using the FXNI151F murine model, we have analyzed for the first time the disturbances to mammalian iron homeostasis caused by systemic partial frataxin deficiency, thus approaching the pathological conditions experienced by FA patients. We have focused on the cerebellum, heart, and liver. The cerebellum and heart are among the most affected tissues in FA, while the liver has a crucial role in systemic iron homeostasis. It is worth mentioning that the studies conducted to date do not provide sufficient evidence to determine whether patients carrying the I154F mutation display clinical features distinct from those of GAA homozygotes. In a study analyzing the clinical features of 25 patients with identified frataxin point mutations, the three patients presenting the I154F mutation did not present cardiomyopathy.^36^ Nevertheless, previous studies had concluded that the clinical picture of patients carrying the I154F mutations was indistinguishable from that of patients homozygous for the expansion reported, and that most I154F patients presented cardiomyopathy.^37^^,^^38^ Without a larger number of patients examined, it cannot be concluded that the clinical presentation of these patients is different from that of the homozygotes.

The obtained results indicate that alterations in iron homeostasis are age-dependent and tissue-specific, with early iron accumulation in the cerebellum and a later accumulation in the liver of female mice. In the heart, where total iron content is not altered, IRP2 content and *Tfrc* expression are increased, while FT-H is decreased. Similar alterations had previously been described in the hearts of cardiac KO mice (MCK mice).^14^ However, two differences exist between both models: (1) iron accumulated in the MCK mice, while in the I151F model, total iron content is not altered, and (2) decreased ACO2 content was reported in the MCK mice, while we have observed decreased ACO2 activity without loss of ACO2 protein content. These differences are likely attributable to the distinct degree of frataxin deficiency exhibited by each model. In this sense, our results demonstrate that under partial pathological frataxin deficiency, the heart shows an iron deficiency response. The observation that the reduction in ACO2 activity is not accompanied by a loss of protein content points to the loss of its iron-sulfur cluster. This loss may result from a decreased rate of iron-sulfur cluster biogenesis, as frataxin has been shown to facilitate this process by accelerating the transfer of persulfide from NFS1 to ISCU (reviewed in Monfort et al.^39^). Nevertheless, decreased iron-sulfur biogenesis could also be caused by limited iron availability due to the formation of iron complexes or aggregates (which would render this metal unavailable for the iron-sulfur biogenesis machinery, causing a paradoxical iron deficiency condition). Such anomalous iron complexes or aggregates have been observed in the hearts of the MCK mice^14^ and in frataxin-deficient yeast.^40^ Uncovering the precise mechanism causing ACO2 deficiency in 21-week-old hearts is beyond the objectives of the present work. Nevertheless, the absence of ACO2 deficiency at 10-week of age supports the hypothesis of limited iron availability for iron-sulfur biogenesis, as decreased iron-sulfur biogenesis rate should have a similar impact on ACO2 activity at younger ages. Regarding the causes of IRP2 accumulation, as previously noted, this phenomenon has also been observed in other models of the disease, including the MCK mouse. Several authors have proposed that such activation may result from a cytosolic iron deficiency, presumably caused by the accumulation of this metal within mitochondria. Nevertheless, while this hypothesis is plausible, it has never been conclusively demonstrated. In fact, multiple studies have reported that cytosolic iron levels are not reduced in yeast,^29^ fibroblasts,^15^ or frataxin-deficient mice.^11^ Our own fractionation experiments (Figure S2) support this view. Thus, IRP2 accumulation does not appear to arise from a deficit in cytosolic iron but rather from a reduction in the biosynthesis or availability of iron-sulfur centers, which are known to participate in IRP2 regulation.

Contrary to the heart, the alterations found in the cerebellum and liver cannot be explained by activation of the IRP/IRE regulatory system. In these tissues IRP1 activity is decreased (according to EMSA assays) and IRP2 content is not significantly altered. Such decreased IRP1 activity should lead to low *Tfrc* mRNA levels and high ferritin content. Although low IRP1 activity could explain the increase in ferritin levels observed at 21-weeks (in both tissues), *Tfrc* mRNA levels are not significantly altered at this age. Moreover, IRP1 activity is already reduced at 10 weeks of age, a stage at which ferritin content is not elevated, or is even decreased (in the liver). Taken together, these observations indicate that the detected alterations do not appear to result from disruption of the IRP/IRE pathway and suggest that other regulatory systems may be altered in frataxin-deficient cerebellum and liver. In this regard, it has been described that *Tfrc* and ferritins expression can be modulated by multiple systems, such as Regnase-1,^41^ Roquin,^42^ tristetraprolin,^43^ NCOA4,^44^ MTF1,^45^ Nrf2 or by microRNAs (reviewed in Galy et al.^16^). Thus, future research should explore the potential implication of these senso-regulatory systems in the iron alterations found in frataxin-deficient cerebellum and liver.

We have observed decreased ACO1/IRP1 protein content in the liver and the cerebellum, but not in the heart. Loss of this protein had previously been observed in cellular^46^ and animal^17^^,^^18^ frataxin KO models and attributed to impaired iron-sulfur biogenesis. In our model, iron-sulfur cluster loss could partially explain the ACO1/IRP1 deficiency in the cerebellum, where decreased content and activity of ACO2 is observed and an increase in the proportion of active IRP1 is detected (potentially caused by iron-sulfur cluster loss). However, these events are not observed in 10-week-old liver, indicating that iron-sulfur biogenesis is not severely affected in this tissue. In this context, it is noteworthy that previous studies using conditional liver frataxin KO animals had already noticed that ACO1 was more strongly affected than the mitochondrial isoenzyme ACO2.^17^ Our results further support this greater susceptibility of the cytosolic isoenzyme to frataxin deficiency and suggest that additional mechanisms (beyond iron-sulfur cluster loss) may contribute to ACO1/IRP1 deficiency in the liver. These mechanisms should be post-translational, as we have not observed differences in ACO1/IRP1 mRNA expression levels. In this regard, frataxin could be directly involved in ACO1/IRP1 stability, as it has been suggested that an extramitochondrial form of frataxin interacts with ACO1/IRP1 and regulates the switch between its both proteoforms.^47^ It should be noted, however, that the existence of a cytosolic frataxin proteoform remains a matter of debate. Moreover, decreased ACO1/IRP1 protein content could be caused by iron toxicity or oxidative stress, as low levels of this protein have also been reported in other conditions where iron or oxidative stress are altered, such as liver biopsies from hereditary hemochromatosis patients,^48^ in macrophages treated with paraquat^49^ and in livers of SOD1-deficient mice.^50^ In addition, the observed phosphorylation of IRP1 at Ser-138 suggests that its deficiency could also result from alterations in regulatory pathways. In this regard, this protein can be regulated by the MEK/ERK and PI3K/Akt pathways^51^ and phosphorylated by protein kinase C.^34^ Indeed, there is evidence indicating that frataxin deficiency causes alterations in the AKT^18^^,^^52^ and in the LKB1/AMPK pathways,^25^ as well as marked metabolic remodeling in both budding and fission yeasts.^53^^,^^54^^,^^55^ Dysregulation of regulatory pathways would explain the tissue and age specificity observed, as signaling networks show high specificity. In other words, if ACO1/IRP1 loss was exclusively caused by impaired iron-sulfur cluster biogenesis, this phenomenon would occur in all conditions analyzed, irrespective of age or tissue, and would affect most iron-sulfur proteins.

Despite being well-established that frataxin deficiency disturbs iron homeostasis, the specific role of iron in the pathogenesis of FA remains unclear. Iron-mediated toxicity has been reported in several models of the disease.^56^ However, symptoms or markers commonly associated with iron deficiency are also found in FA patients, such as serum ferritin values in the lower range or restless leg syndrome,^57^ a clinical condition associated with iron depletion.^58^ Moreover, IRP1 activation (which promotes iron uptake) has a protective effect in a mouse model of hepatic frataxin deficiency,^17^ while dietary iron supplementation ameliorates cardiac hypertrophy in the cardiac conditional frataxin KO mutant mice.^14^ Iron chelation therapies have provided mild beneficial effects at low doses, but a worsening of the condition at higher doses of the drug.^59^^,^^60^ Overall, these findings suggest that the pathological mechanisms in FA could be related to both iron accumulation and limited iron availability. In this context, the results obtained in the present work demonstrate that pathological frataxin deficiency in mice affects iron homeostasis in a tissue-specific manner. This tissue specificity could be behind the contradictory observations regarding the role of iron in FA. Our findings offer valuable insights into this specificity, which may contribute to the design of therapeutic interventions that aim to improve FA symptomatology.

### Limitations of the study

In this work, we clearly demonstrate that partial frataxin deficiency results in a reduction of IRP1 levels in both the cerebellum and the liver. Our findings also suggest that impaired iron-sulfur cluster biogenesis is not the sole contributing factor. However, we were unable to definitively identify the additional mechanisms causing this decrease. Furthermore, our data indicate that in the cerebellum and liver, alterations in the expression of ferritins and *Tfrc* may be associated with regulatory processes that are not directly mediated by IRPs. Nevertheless, these processes remain unidentified and further research will be necessary in order to characterize them.

## Resource availability

### Lead contact

Further information and requests for resources and reagents should be directed to and will be fulfilled by the lead contact, Jordi Tamarit (jordi.tamarit@udl.cat).

### Materials availability

The study did not generate new unique reagents or models.

### Data and code availability

• All data generated and analyzed in this study are presented in the figures and supplementary materials and have additionally been deposited in a publicly accessible, citable repository (Mendeley Data: https://doi.org/10.17632/vbcgshjknh.2).
• Any additional information required to reanalyze the data reported in this paper is available from the lead contact upon request.
• No new code was generated in this study.

## Acknowledgments

We thank Roser Pané for technical assistance, as well as the Proteomics and the Elemental Analysis Services from 10.13039/501100009410Universitat de Lleida, the Animal facilities from Universitat de Lleida, and the Lipidomics services from IRBLleida. We also thank Dr. Bruno Galy (Heidelberg) for providing samples from IRP2 KO mice. This study was supported by grant PID2020-118296RB-I00 from MCIN/10.13039/501100011033AEI/10.13039/501100011033 (to J.R. and J.T.); grant PID2023-148128OB-I00 funded by MICIU/10.13039/501100011033AEI/10.13039/501100011033 and by 10.13039/100000015FEDER, UE (to J.T. and E.C.); and grant PID2021-122436OB-I00 from MCIN/10.13039/501100011033AEI/10.13039/501100011033/ERDF “A way to make Europe” (to M.S.), and by Association Française de l'Ataxie de Friedreich – AFAF (to F.D.).

## Author contributions

M.P.-G. and M.M.-C. performed most of the experiments. A.S.-A., M.P.-C., F.D., and L.O.-J. assisted in the collection and preparation of mouse tissues. E.C., G.H., and M.S. assisted and supervised in the biochemical analyses. All authors provided technical support and suggestions for the project and for the manuscript. J.R. and J.T. conceived the project and supervised the study. M.P.-G., M.M.-C., and J.T. designed the experiments, analyzed and interpreted data, and wrote the manuscript.

## Declaration of interests

The authors declare no competing interests.

## STAR★Methods

### Key resources table

**Table:** 

REAGENT or RESOURCE	SOURCE	IDENTIFIER
**Antibodies**		

Anti-Frataxin antibody [EPR21840]	Abcam	Cat# ab219414
Anti-Aconitase 1/ACO1 antibody [EPR7226(2)]	Abcam	Cat# ab183721
Anti-Ferritin heavy chain antibody [EPR3004Y]	Abcam	Cat# ab75973; RRID:AB_1310222
Anti-Ferritin Light Chain antibody	Abcam	Cat# ab69090; RRID:AB_1523609
Anti-ADX antibody [EPR4629]	Abcam	Cat# ab108257; RRID:AB_10862209
Anti-Ndufs1 antibody [EPR11521(B)]	Abcam	Cat# ab169540; RRID:AB_2687932
Anti-POLD1 antibody [EPR15118]	Abcam	Cat# ab186407; RRID:AB_2921290
OxPhos Human WB Antibody Cocktail - 458199	ThermoFisher	Cat# 458099; RRID:AB_2533835
GLRX3 Polyclonal antibody	Proteintech	Cat# 11254-1-AP; RRID:AB_2110374
FAM96A Polyclonal Antibody - PA5-113382	ThermoFisher	Cat# PA5-113382; RRID:AB_2868115
PPAT Polyclonal Antibody - PA5-27770	ThermoFisher	Cat# PA5-27770; RRID:AB_2545246
DPYD Antibody (F-8)	Santa Cruz Biotechnology	Cat# sc-376681; RRID:AB_11150846
IRP2 Antibody	Novus Biologicals	Cat# NB100-1798; RRID:AB_10000490
Goat anti-Rabbit IgG (H + L) Secondary Antibody, HRP - 31460	ThermoFisher	Cat# 31460; RRID:AB_228341
Goat anti-Mouse IgG (H + L) Secondary Antibody, HRP - 31430	ThermoFisher	Cat# 31430; RRID:AB_228307

**Chemicals, peptides, and recombinant proteins**		

I-Block	ThermoFisher	T2015
PVDF membranes	Millipore	IPVH00010
Nitrocellulose membranes	Sigma-Aldrich	10600093
PageRuler™ Plus Prestained Protein Ladder	ThermoFisher	26619
SOLu-Trypsin	Sigma-Aldrich	EMS004
Isocitrate Deehydrogenase	Sigma-Aldrich	I2516
SpikeTidesTM_L	JPT PeptideTechnologies	–
Iodoacetamide	Sigma-Aldrich	I-1149
Acetyl Coenzyme A	Sigma-Aldrich	A2056
Sodium Citrate	Sigma-Aldrich	S4641
Nicotinamide Adenine dinucleotide Phosphate(NADP+)	Sigma-Aldrich	N0505
Oxalacetic acid (OAA)	Sigma-Aldrich	O4126
Bathophenanthrolinedisulfonic acid disodium salt hydrate	Sigma-Aldrich	B1375
Sodium-L-ascorbate	Sigma-Aldrich	A7631
Nitric Acid	ThermoFisher	A509-P1
Hydrogen Peroxide	Scharlau	HI01361000
Dithiobis (2-nitrobenzoic acid)-5,5	Sigma-Aldrich	D8130
30% Acrylamide/Bis Solution, 37.5:1	Bio-Rad	1610158
Triton X-100	Sigma-Aldrich	X100

**Critical commercial assays**		

Immobilon western chemiluminescent hrp substrate	Millipore	WBKLS0500
Pierce^TM^ BCA Protein Assay Kit	ThermoFisher	23225
iScript cDNA Synthesis Kit	Bio-Rad	1708891
TaqMan® 2X Universal PCR Master Mix	Applied Biosystems	4304437
Tfrc1 TaqMan probe	Applied Biosystems	Mm00441941_m1
Aco1 TaqMan probe	Applied Biosystems	Mm00801417_m1
GAPDH TaqMan probe	Applied Biosystems	Mm99999915_g1
Rpl19 TaqMan probe	Applied Biosystems	Mm02601633_g1
TRIzol Reagent	ThermoFisher	15596026

**Experimental models: Organisms/strains**		

C57BL/6J-Fxnem10(T146T,I151F)Lutzy/J	Jackson Laboratory	31922

**Deposited data**		

Western blot, qPCR, Iron content, activities and mass spectrometry raw data	This paper	Mendeley Data: https://doi.org/10.17632/vbcgshjknh.2; https://data.mendeley.com/datasets/vbcgshjknh/2

**Oligonucleotides**		

FAM-FTH-1 IRE RNA probe 5′-UCCUGCUUCAACAGUGCUUGGACGGAAC-3′	GenScript Biotech	Custom

**Software and algorithms**		

Image Lab (versión 6.1.0)	Bio-Rad Laboratories	https://www.bio-rad.com/
Bio-Rad CFX Manager 3.1	Bio-Rad Laboratories	https://www.bio-rad.com/
Skyline	MacCoss Lab Software	https://skyline.ms/

### Experimental model and study participant details

All animal experiments were performed according to the National Guidelines for the regulation of the use of experimental laboratory animals issued by the Generalitat de Catalunya and the Government of Spain (article 33.a 214/1997), which comply with the ARRIVE guidelines. The experimental protocols were evaluated and approved by the Experimental Animal Ethical Committee of the University of Lleida (Approval number CEEA 09-01/24). All procedures were performed at the animal facility*.* FXNI151F heterozygous mice (C57BL/6J-Fxnem10(T146T,I151F)Lutzy/J) were obtained from the Jackson Laboratory, Bar Harbor, ME, USA (Stock Number 31922) as previously described.^23^ Intercrosses of heterozygous animals were performed to generate homozygous WT and FXNI151F mice. Animals were housed in standard ventilated cages with 12 h light/dark cycles and fed with a regular chow diet *ad libitum*. Genotyping was performed by sequencing the *Fxn* gene PCR product amplified from DNA extracted from tail biopsy specimens as previously described.^23^ The experiments were carried out with animals aged 10 and 21 weeks, of both sexes, as indicated in figures and text.

### Method details

#### Western blot

Between 20 and 100 mg of tissue were minced into 2–3 mm^2^ pieces and placed in 1.5 mL screw cap polypropylene tubes in the presence of lysis buffer consisting of 50 mM tris(hydroxymethyl)aminomethane (Tris) HCl pH 7.5, protease inhibitor cocktail and PhosphoSTOP (Roche). 375 μL of lysis buffer per 100 mg of tissue was used. The mixture was homogenized in a BioSpec Mini-Beadbeater with Glass beads (0.5–1.0 mm) After, SDS was added to the mixture at 4% final concentration. This homogenate was vortexed for 1 min, heated at 98°C for 5 min, sonicated and centrifuged at 14000rpm for 10 min. Protein content in the supernatant was quantified using the Pierce BCA Protein Assay Kit (ThermoFisher Scientific) according to the manufacturer’s instructions. After SDS-polyacrylamide gel electrophoresis, proteins were transferred to PVDF (Millipore, IPVH00010) or Nitrocellulose (Sigma_Aldrich, 10600093) membranes and blocked with 3% I-block (ThermoFisher, T2015) for at least 1 h. Membranes were probed with the following primary antibodies overnight: Frataxin 1:1000 (AbCam, ab219414), IRP1/ACO1 1:10000 (AbCam ab183721), H-FT 1:1000 (AbCam, ab75973), L-FT 1:10000 (abCam, ab69090), ACO2 (Sigma, HPA001097), FDX1 1:1000 (AbCam, ab108257), NDUFS1 1:60000 (AbCam, ab169540), OxPhos Rodent Antibody Cocktail 1:3000/1:40000 (Invitrogen, 458099) for SDHB, UQCR2 and ATPA5A detection, POLD1 1:10000 (AbCam, ab186407), GLRX3 1:1000 (Proteintech, 11254-1-AP), DPYD 1:1000 (Santa Cruz, sc-376681), FAM96A (ThermoFisher, PA5-113382), PPAT 1:1000 (LifeTechnologies, PA5-27770) and IRP2 1:1000 (Novus Biologicals, NB100-1798). For IRP2, as this antibody cross-reacts with other neighboring bands, specificity was verified in samples from IRP2 KO mice kindly provided by Dr. Bruno Galy (Heidelberg). Proteins were detected after incubating with peroxidase conjugated secondary antibodies for 1 h and 5min with Immobilon^R^ Western (Millipore) after 5x washes. Images were acquired by ChemiDoc MP system from Bio-Rad. Membranes were stained with Coomassie brilliant blue (CBB) or Ponceau for normalization. When required, data was analyzed using ImageLab software (Bio-Rad). Apparent molecular weights from the analyzed bands were calculated using PageRuler Plus Prestained Protein Ladder (ThermoFisher, 26619).

#### ACO2/CS protein ratio quantification by SRM-proteomics

Tissue homogenates obtained as described above (100 μg of protein) were precipitated with cold acetone (9 volumes) and resuspended in 1% sodium deoxycholate, 50 mM ammonium bicarbonate. Then, proteins were subjected to reduction by 5.3 mM DTT and alkylation by 26 mM iodoacetamide. Proteins were digested overnight at 37°C with mass spectrometry grade trypsin (SOLu-Trypsin, Sigma) in an enzyme:substrate ratio of 1:50. After, formic acid was added to precipitate sodium deoxycholate. The resulting peptide mix was purified and enriched using 100ul Pierce C18 ZipTips. Eluted fraction from the C18ZipTip was evaporated using a Concentrator Plus (Eppendorf) and peptides were resuspended in 3% acetonitrile plus 0,1% formic acid containing heavy peptide standards from ACO2 (NAVTQEFGPVPDTAR and DLEDLQILIK) and CS (GLVYETSVLDPDEGIR and DYIWNTLNSGR) obtained from JPT (SpikeTidesTM_L). All peptide samples were analyzed on a triple quadrupole spectrometer (Agilent 6420) equipped with an electrospray ion source. Chromatographic separations of peptides were performed on an Agilent 1200 LC system using a Supelco Bioshell A160 Peptide C18 column (1 mm × 15 cm). Peptides (up to 15 μg of protein digest) were separated with a linear gradient of acetonitrile/water, containing 0.1% formic acid, at a flow rate of 50 μL/min. A gradient from 3 to 60% acetonitrile in 45 min was used. The mass spectrometer was operated in multiple reaction monitoring mode. Transitions were obtained either from peptide atlas, SRM atlas or Prosit^61^ imported into Skyline software^62^ and validated with the heavy internal standards. Skyline was also used to analyze results. For calculating the ACO2/CS protein abundance ratio, the light to heavy (L/H) ratio obtained for each peptide in each replica was divided by the mean average L/H value of each peptide among all samples and replicas to obtain a normalized L/H value. The normalized L/H value from the different peptides corresponding to the same protein and sample was averaged, and finally sample ACO2/CS ratio was calculated.

#### Enzyme activities

Spectrophotometric analysis of Aconitase/citrate synthase activity ratios was performed as previously reported.^23^ Briefly, tissues were minced into 2–3 mm^2^ pieces and placed into tubes containing non-denaturing lysis buffer consisting of 50 mM Tris-HCl at pH 7.4, protease inhibitor cocktail (Roche) and 2.5 mM sodium citrate. A BioSpec Mini-Beadbeater was used for tissue homogenization with Glass beads (0.5–1.0 mm). Then, Triton X-100 was added at a final concentration of 0.5%. Homogenized tissues were centrifuged for 5 min at 13000 rpm at 4°C and the supernatants were obtained. Aconitase activity was measured in 50 mM Tris-HCl at pH 7.4, containing 1 mM of sodium citrate, 0.2 mM of NADP, 0.6 mM of manganese chloride and 0.25 units of isocitrate dehydrogenase (Sigma-Aldrich, I2002). NADPH formation was measured at 340 nm for 120s. Citrate synthase activity was measured with a coupled assay to reduce 5,5′-dithiobis-(2-nitrobenzoic acid) (DTNB). Briefly, tissue extracts were added to Tris-HCl 100 mM pH 8.1 with 0.4 mg/mL of DTNB and 10 mg/mL of Acetyl-CoA. Absorbance was measured at 412 nm during 120 s. Then, 8.5 mg/mL of oxaloacetate was added into the cuvette and the absorbance was measured again at 412 nm for 120 s for the detection of reduced DTNB. Values are presented as a ratio of aconitase activity versus citrate synthase activity. In-gel aconitase activity analysis was performed as described before^63^ with some modifications. Tissue homogenates were prepared as above (in this case with 50 mM Tris-HCl pH 8.5). 20 μg of total protein was loaded on a gel composed of a separating gel containing 6% acrylamide, 132 mM Tris base, 66 mM borate, 3.6 mM citrate, and a stacking gel containing 4% acrylamide, 66 mM Tris base, 33 mM borate, and 3.6 mM citrate. The running buffer contained 25 mM Tris (pH 8.3), 96 mM glycine, and 3.6 mM citrate. Electrophoresis was carried out at 170 V at 4°C for 2h 45min. After, gels were incubated for 10–15 min at 37°C, dark conditions, in 10 mL of a 100 mM Tris-HCl pH 8 solution containing 1 mM NADP, 2.5 mM *cis*-aconitic acid, 5 mM manganese chloride, 0.3 mM phenazine methosulfate and 2.6 U/ml of isocitrate dehydrogenase until blue bands were observed. Images were acquired in a GelDoc EZ imager (Bio Rad) and band intensity measured by ImageLab software. Relative aconitase activity was quantified by comparison with a standard curve prepared by loading different amounts of WT homogenates on gels.

#### Tissue iron quantification

Tissue non-heme iron was measured using the iron chelator BPS (bathophenanthroline disulfonic acid) after acid-digestion of the sample. A tissue fragment of approximately 30 mg was dried in a SpeedVac at 60°C and weighed. To this sample, 50 μL of MilliQ water and glass beads (diameter between 0.5 and 1 mm, Sigma G8772) were added, and tissue was homogenized using a BioSpec Mini-Beadbeater. Then, 450 μL of concentrated nitric acid was added, and the mixture was incubated for 2 h at 98°C in a Thermomixer comfort (Eppendorf) before a 5 min centrifugation at 10,000 rpm. Iron in the supernatant was measured using the iron chelator BPS (bathophenanthroline disulfonic acid) as previously described.^64^ Supernatant (400 μL) was mixed with 160 μL 38 mg/mL sodium ascorbate, 320 μL 1.7 mg/mL BPS, and 126 μL ammonium acetate solution (saturated ammonium acetate diluted 1/3). After 5 min, the specific absorbance of the iron–chelator complex was recorded at 535 nm in a Shimadzu UV-2401 PC spectrophotometer. Nonspecific absorbance was recorded at 680 nm and subtracted. Blanks and standards (prepared with ferrous ammonium sulfate) were also analyzed to prepare standard curves and correct for potential iron contamination in the materials and reagents used.

#### Measurement of mitochondrial and cytosolic iron

Hearts (WT or FXNI151F) were collected and processed immediately using ice-cold reagents to minimize mitochondrial degradation. All solutions were adjusted to pH 7.4. Tissues were washed with mitochondrial isolation buffer (10 mM Na-HEPES, pH 7.4, 300 mM sucrose, 200 μM EDTA, supplemented with protease inhibitors [Roche, 04693159001] and phosphatase inhibitors [Roche, 04906845001]). Hearts were minced into small pieces and incubated for 10 min at 4°C in 10 mL isolation buffer containing 0.1 mg/mL trypsin, followed by addition of 10 mL isolation buffer containing 0.5 mg/mL trypsin inhibitor (Merck, T6522). Tissues were centrifuged at 800 × g for 3 min at 4°C, and the pellets were resuspended in 8 mL isolation buffer. Homogenization was performed on ice using a motor-driven Teflon Potter homogenizer (Glass Col 099C K54) with 6–7 strokes, avoiding bubble formation. Homogenates were centrifuged at 800 × g for 10 min at 4°C to remove nuclei and debris, and the supernatant was transferred to 1.5 mL tubes. Samples were centrifuged at 8000 × g for 15 min at 4°C to pellet mitochondria. Supernatant was considered the cytosolic fraction. Mitochondrial pellets were combined, resuspended carefully, and centrifuged again at 8000 × g for 15 min at 4°C. The final mitochondrial pellet was resuspended in 100–150 μL isolation buffer. Protein concentration was determined using the Bradford assay (Biorad, 5000006) according to the manufacturer’s instructions. Iron analysis was conducted as described,^65^ with some modifications. Cytosolic or mitochondrial fractions were dissolved in 0.4 mL of concentrated nitric acid (HNO_3_) and incubated at 80°C for 4h in closed screw-capped tubes. To remove lipids, samples were cooled to room temperature, tubes opened and 0.4 mL 30% hydrogen peroxide added. After 10 min at room temperature the tubes were closed again and incubated at 70°C for 1h. Once cooled, samples were diluted in milliQ water and Fe levels measured by inductively coupled plasma mass spectrometry (ICPMS).

#### Quantitative real-time PCR

For qRT-PCR analysis, 50–100 μg of tissue samples were homogenized with 1 mL of TRIzolTM Reagent (ThermoFisher, 15596018) using an IKA T10 basic homogenizer. RNA was extracted following the manufacturer’s instructions. For each sample, 1 μg of total RNA was converted into cDNA with iScript cDNA Synthesis Kit (Bio-Rad, 1708891) and 50 ng of cDNA was used in each reaction. Assays were performed in a CFX96 Real-Time System (Bio-Rad) using TaqMan 2X Universal PCR Master Mix (Applied Biosystems, 4304437) mixed with TaqMan probes: Tfrc1 (Mm00441941_m1), Aco1 (Mm00801417_m1) and GAPDH (Mm99999915_g1) or Rpl19 (Mm02601633_g1) as a loading control. Quantification was completed using Bio-Rad CFX Manager real-rime detection system software (version 3.1, Bio-Rad). Relative expression ratios were calculated based on ΔCp values with efficiency correction considering multiple samples.

#### EMSA assay

Cytosolic extracts were isolated from liver (1–2 mm^3^ pieces) in 0.5 mL of ice-cold cytoplasmatic lysis buffer (CLB) buffer (25 mM Tris-HCl pH = 7,4, 40 mM potassium chloride, 1% Triton X-100, 1 mM DTT and Complete EDTA free protease inhibitor cocktail (Roche), with a Dounce tissue homogenizer for 10 s. After chilling on ice for 20min and centrifuge for 10min full speed at 4°C, supernatants corresponding to cytosolic fractions were obtained. Then, the protein was quantified by Bio-Rad protein assay (ref. 5000006) and 25 μg of total protein was mixed with CLB up to 10 μL and 1 μM FAM-labeled FTH-1 IRE probe previously heated for 1 min at 95°C. The FAM-labeled fluorescent FTH-1 IRE probe with the sequence 5′-UCCUGCUUCAACAGUGCUUGGACGGAAC-3′ was prepared by GenScript Biotech (Netherlands). β-mercaptoethanol was added before the probe to the corresponding positive controls for total IRP binding activity. After 20min incubation, a 10-min incubation with 1 μL of 50 mg/ml heparin was done, both at room temperature in dark conditions. Components were separated using 5% TBE gels with a 1:60 acrylamide: bis ratio, at 100V for 45 min. Subsequently, image acquisition was performed in a ChemiDoc MP system from Bio-Rad with blue light epi illumination and a 530/28 emission filter.

### Quantification and statistical analysis

F*or* statistical analyses, GraphPad Prism (version 8) was used. Differences between groups in iron content were assessed by two-way ANOVA with the Tukey’s multiple comparison test when considering more than two categorical variables into the analyses. Two-tailed Student’s *t* test was used to assess the significance of differences between WT and I151F mice in proteins, mRNA expression, EMSA and aconitase activity experiments. Significant *p* values are indicated as <0.05(∗), <0.01(∗∗), <0.001(∗∗∗), <0.0001(∗∗∗∗). The number of mice included in each experiment were indicated as individual points in figures.
