# Supplementary material for: Pathological frataxin deficiency in mice causes tissue-specific alterations in iron homeostasis
Source: iScience. 2026 Jan 5;29(2):114625. doi: 10.1016/j.isci.2025.114625 (PMC12857413; doi:10.1016/j.isci.2025.114625)
Supplement: Document S1. Figures S1–S5 [file mmc1.pdf]

iScience, Volume *29*

Supplemental information

Pathological frataxin deficiency in mice causes tissue-specific alterations in iron homeostasis

Maria Pazos-Gil, Marta Medina-Carbonero, Arabela Sanz-Alcázar, Marta Portillo-Carrasquer, Luiza Oliveira-Jorge, Gonzalo Hernández, Mayka Sánchez, Fabien Delaspre, Elisa Cabiscol, Joaquim Ros, and Jordi Tamarit

Sup. Fig. 1

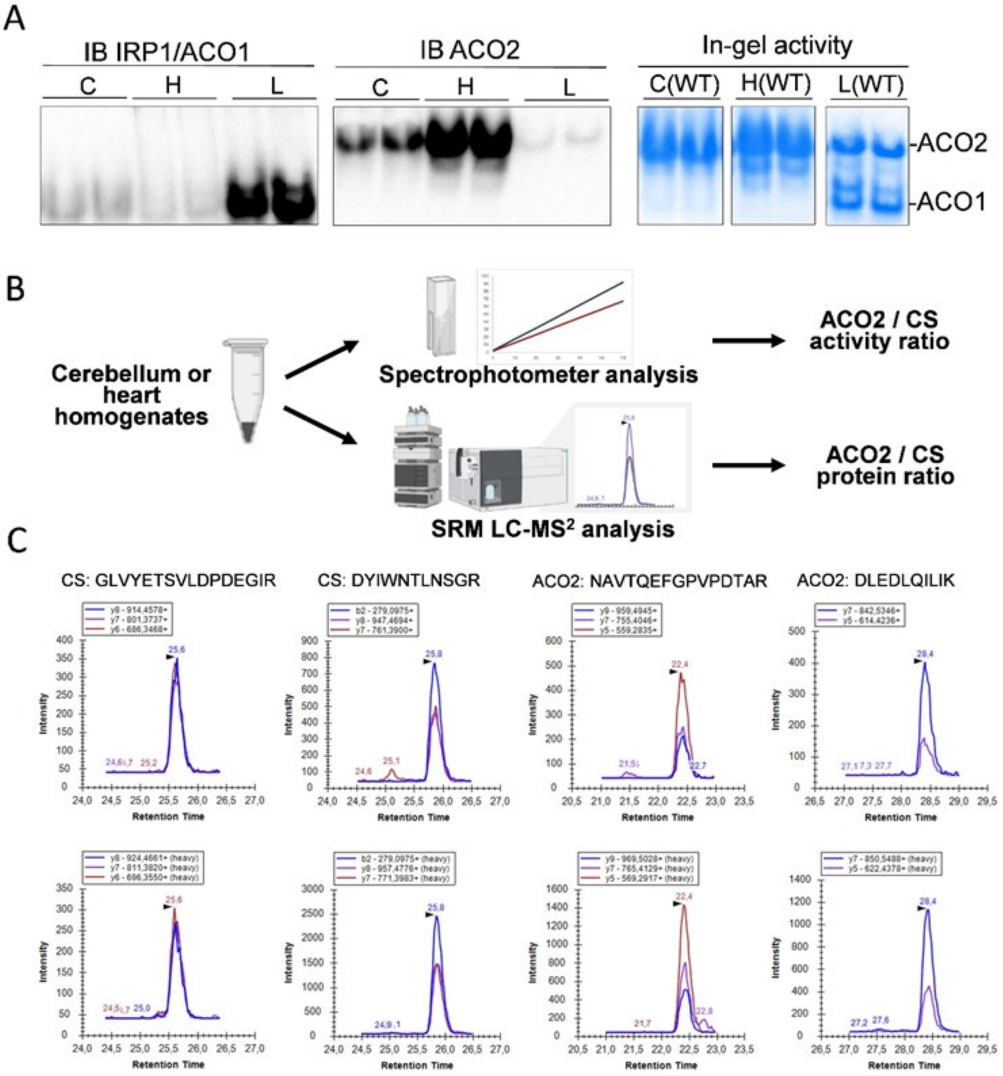

Supplemental Figure 1- Experimental approach used for aconitase assays.

A, Migration of aconitase isoenzymes in native gels used for in-gel aconitase activity. Cerebellum (C), heart (H) and liver (L) homogenates were obtained from WT mice (20 µg for heart and liver, 40 µg for cerebellum) and loaded on native gels and either assayed for aconitase activity or transferred to PVDF membranes for immunoblot (IB) analysis with antibodies against IRP1/ACO1 or against ACO2. Four bands were detected in activity gels. The immunoblot indicates that the two upper bands correspond to ACO2, while the two lower bands correspond to ACO1. This activity was barely detected in the cerebellum and heart, consistent with the low ACO1 IB signal observed in these tissues.

B, Procedure for analyzing Aconitase 2 / Citrate synthase (ACO2/CS) activity and protein ratios from cerebellum and heart homogenates. Spectrophotometer analysis was used to measure activity ratio, while SRM-based LC-MS^2 analysis was used to measure protein ratio.

C, Representative SRM traces used for quantification of ACO2/CS protein ratio. Two peptides from citrate synthase (CS) and Aconitase 2 (ACO2) were analyzed. The upper trace corresponds to the endogenous peptide (light version), while the lower trace corresponds to the internal standard (heavy version). Images correspond to peptides obtained from a 21-week-old WT mice heart sample.

Sup. Fig. 2

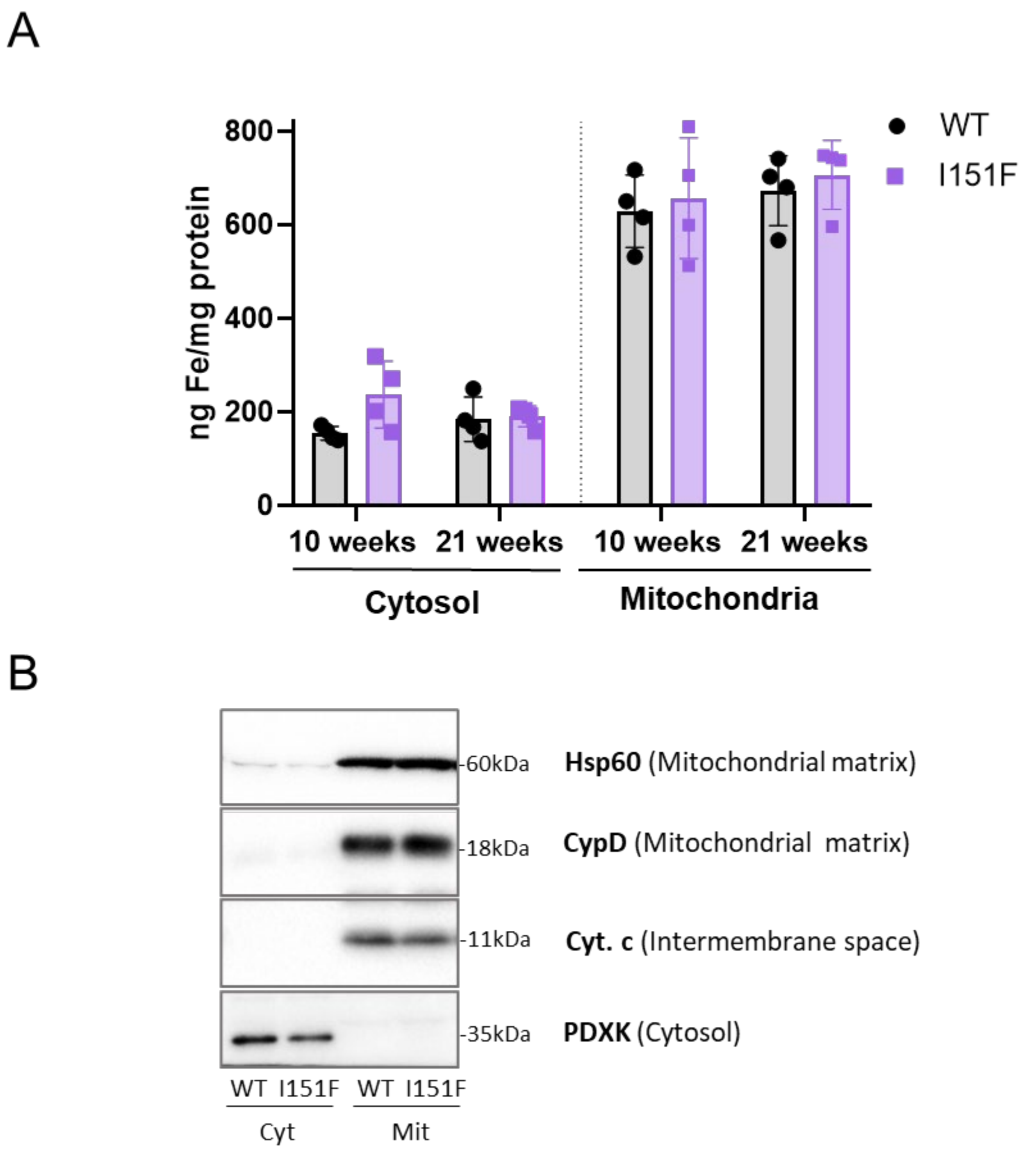

Supplemental Figure 2, Iron content in heart mitochondrial and cytosolic fractions.

A, Iron content measured by ICP-MS in cytosolic and mitochondrial fractions.

B, Representative western blots showing absence of cross-contamination between cytosolic and mitochondrial fractions. The analyzed proteins were Heat Shock Protein 60 (Hsp60), Cytochrom c (Cyt c), Cyclophilin D (CypD) and Pyridoxal kinase (PDXK).

Sup. Fig. 3

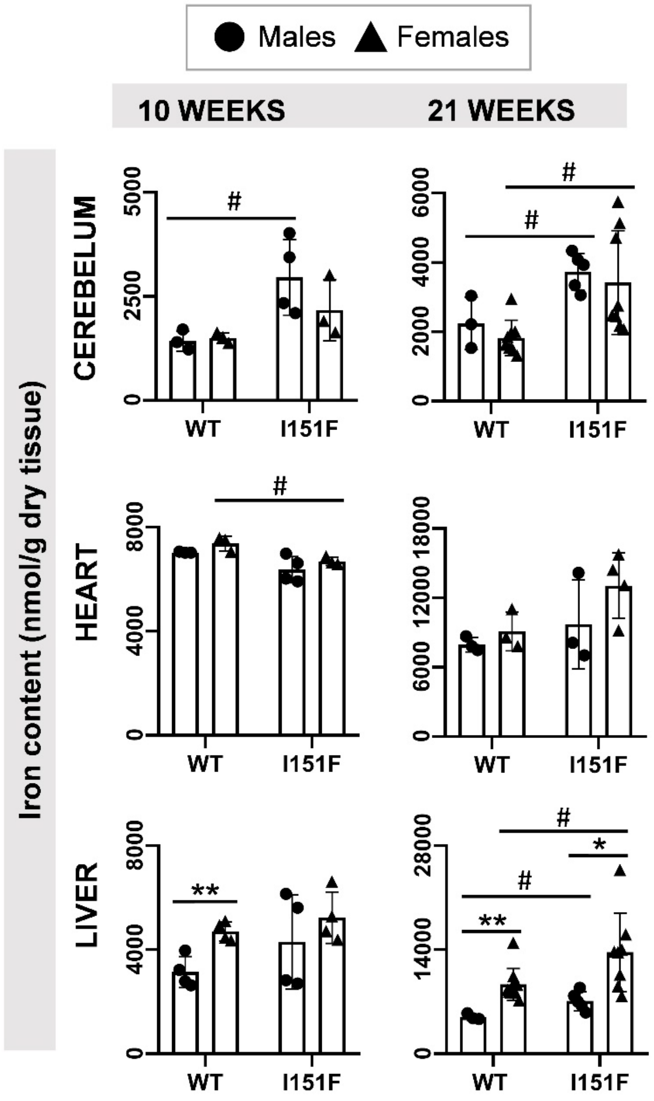

Supplemental Figure 3- Differences in iron content between males and females. Non-heme iron was quantified in the indicated tissues from mice at different ages. Data is represented as mean +/- SD. Significant differences between females and males are indicated by * ($p < 0.05$) or ** $p < 0.01$. Significant differences between WT and I151F are indicated by ♯ ($p < 0.05$).

Sup. Fig. 4

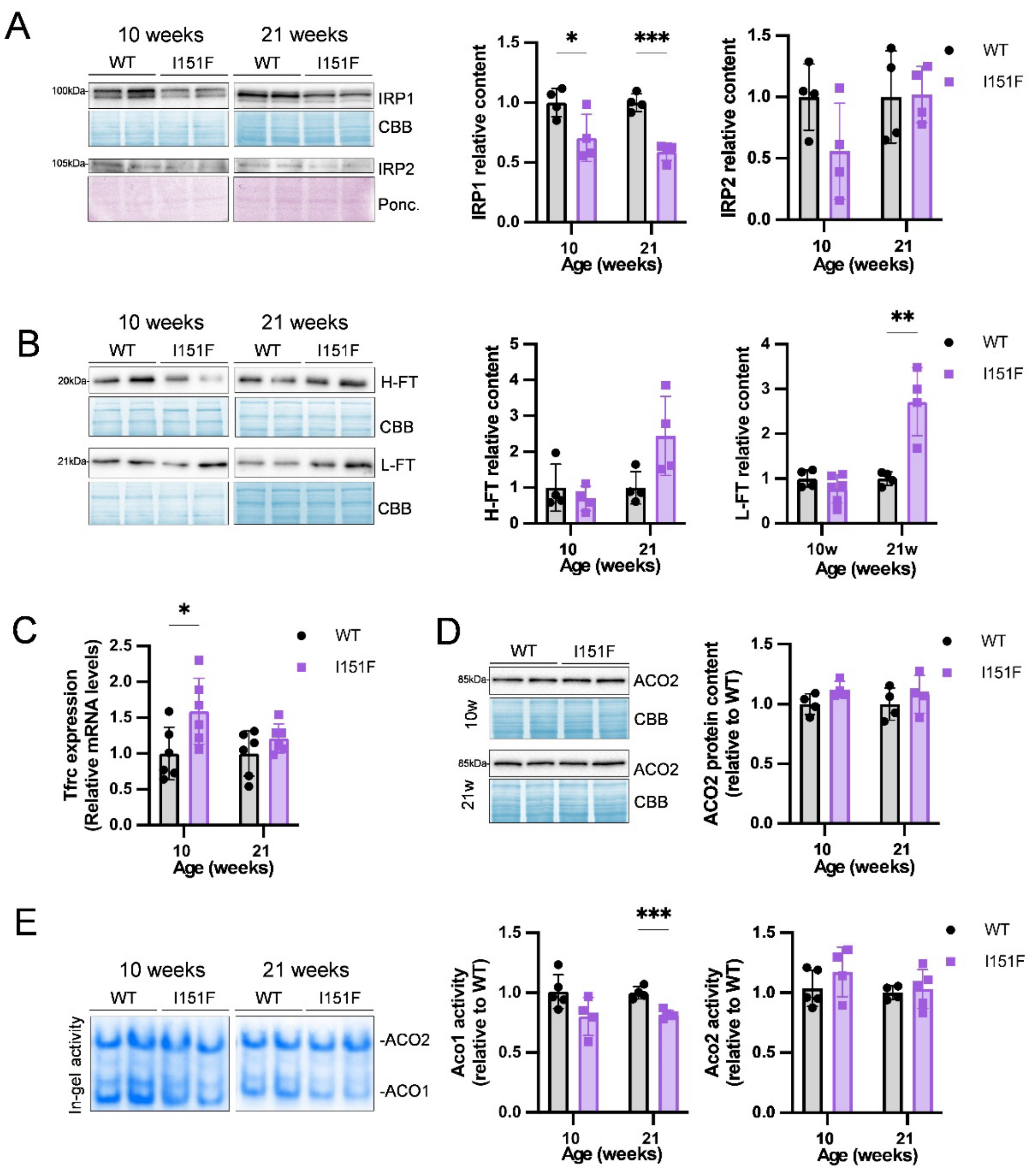

Supplemental Figure 4 - Consequences of frataxin deficiency in male's liver. For all proteins assessed, representative western blot images are shown, while histograms represent the relative protein content calculated from the western blot signal normalized to the CBB or Ponceau stain.

A, ACO1/IRP1 and IRP2 protein content measured by western blot.

B, Ferritins heavy and light chains measured by western blot.

C, Tfrc mRNA content measured by qPCR.

D, ACO2 content measured by western blot.

E, Aconitase 1 and 2 activities measured by an in-gel activity assay.

All quantitative data are represented as mean +/- SD, normalized to WT levels. Individual data values (biological replicates) are indicated as circles (for WT) or squares (for FXNI151F). Significant differences in p-values < 0.05(*), 0.01(**) or 0.001(***), between WT and FXNI151F mice are indicated (t-test analysis).

Sup. Fig. 5

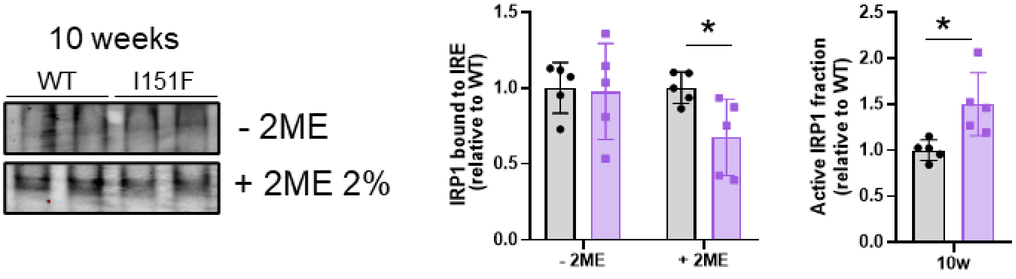

Supplemental Figure 5. Cerebellum EMSA analysis from 10-week-old animals. Left, representative assays showing the band corresponding to the IRP1-bound IRE mRNA probe (IRP1-IRE), in the presence and absence of 2ME. Right, quantification of the IRP1-IRE band and estimation of the active IRP1 fraction (relative to WT) found in cerebellum of 10-week-old females.
